# Comparing Clinical and Radiological Features in Familial and Sporadic Multiple Sclerosis

**DOI:** 10.7759/cureus.44504

**Published:** 2023-09-01

**Authors:** Sena Destan Bunul

**Affiliations:** 1 Neurology, Kocaeli University, Kocaeli, TUR

**Keywords:** ms disability, disability, edss, familial disease, types of multiple sclerosis, multiple sclerosis

## Abstract

Objective

To compare the initial presentation, clinical features, disease courses, and radiological parameters between familial multiple sclerosis (fMS) and sporadic multiple sclerosis (sMS) to determine if the two represent distinct clinical entities.

Methods

This retrospective study was conducted at the Neurology Clinic at Kocaeli University Hospital. Records of 114 fMS and 150 sMS patients, aged 18-65, diagnosed based on either the Poser criteria or the McDonald 2001 criteria were analyzed. Radiological data and Expanded Disability Status Scale (EDSS) evaluations were conducted by a specialist neurologist. Variables included age at MS onset, first symptoms, relapses, EDSS scores at diagnosis and last examination, and MRI findings. Statistical Package for the Social Sciences (IBM SPSS Statistics for Windows, IBM Corp., Version 28, Armonk, NY) was utilized for data analysis.

Results

Both fMS and sMS groups were comparable in age (43.55±12.50 and 42.35±10.61 years, respectively) and gender distribution (females: fMS 71.9%, sMS 71.3%). No significant difference was noted regarding disease onset age (fMS 29.83±10.77, sMS 30.42±9.7). Age of onset, final EDSS, and relapse rate didn't significantly vary among sMS, fMS with first-degree relatives having MS (fMS(1)), and fMS with second or third-degree relatives having MS (fMS(2)). The fMS group showed a significantly higher incidence of initial spinal cord lesions on MRI compared to the sMS group (38.6% vs. 17.3%; p<0.001). Within the fMS group, the presence of spinal cord lesions on initial MRI correlated with a higher relapse rate and elevated initial and final EDSS scores.

Conclusion

Despite overarching similarities between fMS and sMS, spinal cord lesions' prevalence and implications in fMS may point to a genetic underpinning warranting in-depth exploration.

## Introduction

Multiple sclerosis (MS) is considered to be a chronic and neurodegenerative central nervous system disease that develops on an autoimmune background [1]. MS, a demyelinating disease, occurs mostly in genetically susceptible individuals with the influence of environmental factors [2]. MS affects more than 2.8 million people worldwide [3]. Although it does not show Mendelian inheritance, MS, which is known to be associated with certain genes and gene loci, is more common in family members of affected individuals compared to the general population [4]. Numerous genes and their polymorphisms including biomarkers have been investigated for many years as indicators of susceptibility to MS development [5]. Familial multiple sclerosis (fMS) is defined as a case of MS in which at least one first- to third-degree relative has a diagnosis of MS [6]. One study showed that the global prevalence of fMS is approximately 12.6% [7]. However, the prevalence of fMS varies, especially by geographical location. In regions such as North America and Europe, where the prevalence of MS is highest, the incidence of fMS is also higher, while in places such as Asia, where the prevalence of MS is low, the incidence of fMS is correspondingly lower [8-11]. This suggests that the risk of fMS increases with the combined burden of genetic and environmental risk factors [7,12]. When comparing the risk factors and clinical courses of fMS and sporadic multiple sclerosis (sMS), some studies have shown no significant difference between the two forms of MS in terms of clinical clinical presentation. In contrast, others have identified differences in demographics, clinical presentation, and radiological findings between the two groups [1,13-21]. In a study conducted, brain stem and cortical dysfunction were observed more frequently in the fMS group compared to the sMS group. In another study, the initial symptoms of MS showed significant differences between fMS and sMS groups. The primary reason for this was a reduced frequency of double vision in the fMS group [16]. A few studies have shown that genetic factors affect disease progression but are not associated with disease severity [17]. However, there is still insufficient data to determine whether fMS differs from sMS. Our aim of this study was to evaluate the differences between fMS and sMS in clinical onset features, disease course, and some radiological parameters. It is also to illuminate the literature on whether fMS and sMS are distinct clinical entities.

## Materials and methods

This research is a retrospective study conducted at the Demyelinating Diseases Unit of the Neurology Clinic at Kocaeli University Hospital. Data were collected from medical records. Patients aged 18-65 who were clinically diagnosed with definite MS according to the McDonald criteria, including 114 fMS and 150 sMS patients, were included. Immigrants were excluded because of differences in immunogenetic backgrounds. Unrelated fMS patients were selected; in other words, only the index case from each verified related patient pair was included. No other inclusion/exclusion criteria were applied to the case selection. The Expanded Disability Status Scale (EDSS) assessment and interpretation of magnetic resonance imaging (MRI) data were performed by a neurologist experienced in demyelinating diseases. Ethical approval for the study was obtained from the university's local ethics committee. fMS and sMS groups were compared in terms of clinical characteristics such as age at onset of MS, course of first symptom and relapses, EDSS scores at the time of diagnosis and at the last examination, as well as demographic characteristics such as educational level, gender, and age. In addition, both groups were compared regarding the presence or absence of contrast-enhancing lesions in MRI and the presence or absence of spinal lesions.

Statistical Package for the Social Sciences (IBM SPSS Statistics for Windows, IBM Corp., Version 28, Armonk, NY) was used for the analysis of the data included in the study. The Kolmogorov-Smirnov test was used for the given normality test. The chi-square test was used when comparing categorical data, and Fisher's exact test was used when necessary. The Mann-Whitney U test was used to compare two independent groups, the Kruskal-Wallis test was used to compare more than two groups, and the Friedman analysis was used for repeated measurements. The statistical significance level was set as <0.05. Binary logistic regression analysis was used to estimate EDSS.

## Results

In the study, 150 sMS patients with a mean age of 42.35±10.61 (range 23-70) and 114 fMS patients with a mean age of 43.55±12.50 (range 21-74) were evaluated. The gender ratio of females to males was similar in both groups (fMS: 71.9%, sMS: 71.3%). In the fMS group, 56.1% had at least one first-degree relative diagnosed with MS, while 43.8% had at least one second- or third-degree relative diagnosed with MS. There was no statistically significant difference between the groups in terms of age and gender (respectively, p=0.748, p=0.514). There was no statistically significant difference between the sMS and fMS groups in terms of age at disease onset. The ages at disease onset were 30.42±9.7 in the sMS group and 29.83±10.77 in the fMS group, respectively (p=0.625). When the study groups sMS, fMS(1) (group with a first-degree relative having MS), and fMS(2) (group with a second or third-degree relative having MS) were compared using the Kruskal-Wallis test in terms of age of disease onset, initial EDSS, final EDSS, and disease duration: there was no statistically significant difference in onset age (p=0.532), final EDSS (p=0.263), and relapse rate (p=0.294) among the three groups, whereas patient age (p=0.039), initial EDSS (p<0.001), and disease duration (p<0.001) were significantly different (Table 1).

**Table 1:** Comparative Analysis of Clinical and Demographic Parameters among sMS, fMS(1), and fMS(2) Groups sMS: sporadic multiple sclerosis, fMS(1): group with a first-degree relative having MS, fMS(2): group with a second or third-degree relative having MS, EDSS: Expanded Disability Status Scale In the comparative analysis, * represents the comparison between sMS, fMS1, and fMS2 groups; ** denotes the comparison between fMS1 and fMS2; *** signifies the comparison between sMS and fMS1; and **** indicates the comparison between sMS and fMS2.

	sMS group (mean±SD)		fMS(1) group (mean±SD)	fMS(2) group (mean±SD)	p-value
Age of disease onset (years)		30.42±9.70	29.39±10.75	30.40±10.88	0.532^*^
Current age (years)		42.35±10.61	45.95±12.27	40.48±12.23	0.039^*^
Disease duration (years)		12.93±6.52	14.94±7.26	9.54±6.57	<0.001^*^ <0.001^**^ 0.072^***^ <0.001^****^
Initial EDSS		1.49±0.89	1.82±0.74	1.05±0.81	<0.001^*^ <0.001^**^ 0.018^***^ 0.002^****^

When both groups were compared in terms of clinical features, the disease duration was 12.93±6.52 years for the sMS group and 12.57±7.44 years for the fMS group (p=0.612). When the changes in the baseline and final EDSS scores of both groups were evaluated, a significant increase in the final EDSS score was observed in both groups compared with the initial evaluation. However, there was no statistically significant difference between the two groups (p=0.762). Similar to the increase in EDSS score, no significant difference was observed between the two groups in other clinical features summarized in Table 2.

**Table 2:** Comparative Analysis of Clinical Features and EDSS Score between sMS and fMS Group sMS: sporadic multiple sclerosis, fMS: familial multiple sclerosis, EDSS: Expanded Disability Status Scale

	sMS group; (n=150) (mean±SD) (min-max)	fMS group; (n=114) (mean±SD) (min-max)	p-value
Age (years)	42.35±10.61 (23-70)	43.55±12.50 (21-74)	p=0.748
Disease duration (years)	12.93±6.52 (3.39)	12.57±7.44 (1-41)	p=0.612
Relapse rate	3.71±1.37 (2-8)	3.48±1.28 (1-8)	p=0.294
First EDSS score	1.49±0.89 (0-4)	1.48±0.86 (0-4)	p=0.897
Last EDSS score	3.15±1.14 (1-7)	3.18±1.13 (1-7)	p=0.691

When the study group was evaluated based on clinical and radiological onset characteristics, the fMS group had a higher proportion of lesions in the spinal cord on the initial MRI compared to the sMS group (38.6% vs. 17.3%; p<0.001). No significant differences were observed between the two groups regarding other characteristics, with the data provided in Table 3.

**Table 3:** Comparison of Initial Clinical and Radiological Onset Characteristics between fMS and sMS Groups sMS: sporadic multiple sclerosis, fMS: familial multiple sclerosis

	sMS group; (n=150)	fMS group; (n=114)	p-value
Supratentorial onset	37%	35%	0.708
Optic neuritis onset	23.3%	22.8%	0.920
Brainstem onset	36.7%	34.2%	0.680
Transverse myelitis	37.3%	34.2%	0.601
Gadolinium enhancement	20%	24.6%	0.376
Presence of spinal cord lesion	38.6%	17.3%	<0.001

In the sMS group, there was no difference in terms of relapse rate and initial and final EDSS for those with and without spinal lesions on the initial MRI (respectively, p=0.809, p=0.683, p=0.473). In the fMS group, those with spinal lesions on the initial MRI had a higher relapse rate, and both initial and final EDSS scores were found to be higher than those without spinal lesions (respectively; p<0.001, p=0.002, p=0.012).

For both groups in the study, when predicting clinical and radiological factors for an EDSS score of 3 or above at the final evaluation using binary logistic regression analysis, the presence of a spinal cord lesion was a significant predictor in the fMS group, whereas no significant effect of spinal cord lesion presence was identified in the sMS group. In the fMS group, the absence of a lesion in the spinal cord reduced the risk of having an EDSS score of 3 or above by 0.34 times (Table 4).

**Table 4:** Evaluation of Predictive Factors for an EDSS Score of 3 and Above Using Binary Logistic Analysis (Significant Data Provided) fMS: familial multiple sclerosis, EDSS: Expanded Disability Status Scale

	Accuracy	p-value	Odds Ratio	95% C.I. for EXP(B)	
				Lower	Upper
Presence of spinal cord lesion in fMS group	71.9%	0.025	0.341	0.132	0.875

## Discussion

In our study, although no significant differences in clinical and demographic features between the fMS and sMS groups were identified statistically, a higher incidence of initial spinal cord lesions was observed in the fMS group. When the relationship between the presence of spinal cord lesions with relapse rate and EDSS was evaluated, there was no significant correlation in the sMS group, whereas a noteworthy correlation between spinal cord lesions, relapse rate, and EDSS was observed in the fMS group. Moreover, the existence of a spinal cord lesion within the fMS group was indicative of an EDSS score of 3 or above, whereas this characteristic feature did not serve as a predictive factor within the sMS group.

MS is a neurodegenerative disease that can cause disability in both adults and pediatric groups. It arises as a result of the interplay between genetic and environmental factors. The risk of developing MS is higher in family members of MS patients. Similarly, studies have shown that MS families carry more MS risk genes compared with patients with sMS [22]. Identifying the genetic characteristics of MS has the potential to provide crucial insights regarding its etiology and contribute to the development of early diagnosis and rational treatments [23].

The question of whether fMS and sMS delineate separate processes continues to be a topic of discussion. The demographic attributes of both groups have produced diverse outcomes across various studies. In a study by Ceccarelli et al., no differences in clinical or demographic aspects, including disease onset age and gender, were observed between the fMS and sMS groups [1]. In a study by Ebers et al., the age of disease onset in fMS was found to be lower compared to sMS [24]. Another study demonstrated that when the fMS group was subdivided into those with a first-degree familial relationship and those with second- and third-degree relationships, the disease began earlier in patients diagnosed with MS who had a first-degree relationship compared with sporadic cases. However, when fMS cases diagnosed in second- and/or third-degree relatives were added to the analysis, the difference in onset age between fMS and sMS was not significant [14]. In our study, no differences regarding disease onset age, gender, and disease duration were observed either between the fMS and sMS groups or between those with first- and third-degree relatives diagnosed with MS and those with second- and third-degree relatives diagnosed with MS.

In a study comparing the initial clinical symptoms of fMS and sMS groups, motor, sphincter, cognitive, and brainstem findings were found to be higher in fMS patients than in sMS patients [6]. Another study identified that the occurrence of the initial symptom as optic neuritis was less frequent in the fMS group [14]. However, our research did not observe any differences between the two groups concerning initial clinical symptoms. This situation can be attributed to the included patient groups having similar EDSS scores and comparable disease progression. The other potential reason for this could be the heterogeneity of patient groups included in other studies. In our research, the data regarding the progression can be particularly valuable due to the similarity in age, gender, and clinical onset characteristics among our study participants.

The EDSS is the most commonly used parameter to evaluate physical disability. In a study examining the EDSS score between the fMS and sMS groups, the disability score in the fMS group was found to be higher than that in the sMS group [6]. However, our findings are consistent with previous studies that identified no significant difference in EDSS (initial-current) between the two groups [1,17]. In addition, when fMS subgroups were included in the analysis, the initial EDSS score was found to be significantly higher in the group with a first-degree relative with MS compared to the other groups. Therefore, it may suggest that more genes are clustered in the group with first-degree relatives with MS. It was considered a very important finding to draw attention to the genetic aspect of MS. With the progression of neurodegeneration in MS, there is an increase in progression over time, and physical disability becomes permanent. The increase in the EDSS score during the course of MS due to neurodegeneration is of significant importance [25]. When evaluating predictive factors in our study for an EDSS score of 3 or above, the presence of spinal cord lesions in the fMS group was a significant predictor, whereas no significant effect of spinal cord lesion presence was observed in the sMS group. A significant relationship between spinal cord and physical disability has been shown by many studies [26-28], and the same relationship has been shown in the fMS group [14]. Our study is very important in terms of contributing to the poor literature on this subject.

Although it is very important to include MRI in addition to the clinical features of fMS in this study, MRI parameters are not detailed. While acknowledging the limitations of our study, we carefully included both spinal cord and gadolinium (Gd) involvement in our evaluation, as these factors are paramount. Specifically, the presence of a contrast-enhancing lesion serves as a marker for initial disease activation, while spinal cord involvement is crucial for understanding disease progression. That said, it's important to note that our study is retrospective, meaning it might lack some of the predictive strengths of a prospective study. Additionally, the size of our sample may influence the strength and applicability of our findings. By recognizing these constraints, we aim to provide a balanced perspective, allowing for informed interpretations of our results.

## Conclusions

While this research shed light on various aspects of sMS and fMS, one finding of particular predictive importance is the prevalence of spinal cord lesions in the fMS group. This highlights a potential avenue for further investigation into the pathophysiological and genetic underpinnings of fMS. Future studies with a larger and more systematically acquired dataset would provide a more comprehensive insight. In addition to many common points of sMS and fMS, it continues to be important to detail the differences that have a possible genetic basis.

## References

[1] Ceccarelli A, Mifsud VA, Dogar A (2020). Demographic and clinical characteristics of familial and sporadic multiple sclerosis: a single center exploratory study from Abu Dhabi. J Clin Neurosci.

[2] Marrie RA (2004). Environmental risk factors in multiple sclerosis aetiology. Lancet Neurol.

[3] Walton C, King R, Rechtman L (2020). Rising prevalence of multiple sclerosis worldwide: Insights from the Atlas of MS, third edition. Mult Scler.

[4] Patsopoulos NA (2018). Genetics of multiple sclerosis: an overview and new directions. Cold Spring Harb Perspect Med.

[5] Katsavos S, Anagnostouli M (2013). Biomarkers in multiple sclerosis: an up-to-date overview. Mult Scler Int.

[6] Faraji F, Mohaghegh P, Talaie A (2022). Epidemiology of familial multiple sclerosis and its comparison to sporadic form in Markazi Province, Iran. Mult Scler Relat Disord.

[7] Harirchian MH, Fatehi F, Sarraf P, Honarvar NM, Bitarafan S (2018). Worldwide prevalence of familial multiple sclerosis: a systematic review and meta-analysis. Mult Scler Relat Disord.

[8] Balnyte R, Rastenyte D, Vaitkus A, Mickeviciene D, Skrodeniene E, Vitkauskiene A, Uloziene I (2013). The importance of HLA DRB1 gene allele to clinical features and disability in patients with multiple sclerosis in Lithuania. BMC Neurol.

[9] Heinzlef O, Alamowitch S, Sazdovitch V, Chillet P, Joutel A, Tournier-Lasserve E, Roullet E (2000). Autoimmune diseases in families of French patients with multiple sclerosis. Acta Neurol Scand.

[10] Nielsen NM, Westergaard T, Rostgaard K (2005). Familial risk of multiple sclerosis: a nationwide cohort study. Am J Epidemiol.

[11] Goodin DS (2014). The epidemiology of multiple sclerosis: insights to disease pathogenesis. Handb Clin Neurol.

[12] Esposito F, Guaschino C, Sorosina M (2015). Impact of MS genetic loci on familial aggregation, clinical phenotype, and disease prediction. Neurol Neuroimmunol Neuroinflamm.

[13] Steenhof M, Stenager E, Nielsen NM, Kyvik K, Möller S, Hertz JM (2019). Familial multiple sclerosis patients have a shorter delay in diagnosis than sporadic cases. Mult Scler Relat Disord.

[14] Katsavos S, Artemiadis A, Davaki P, Stamboulis E, Kilindireas K, Anagnostouli M (2018). Familial multiple sclerosis in Greece: distinct clinical and imaging characteristics in comparison with the sporadic disease. Clin Neurol Neurosurg.

[15] Steenhof M, Nielsen NM, Stenager E, Kyvik K, Möller S, Hertz JM (2019). Distribution of disease courses in familial vs sporadic multiple sclerosis. Acta Neurol Scand.

[16] Andrijauskis D, Balnyte R, Keturkaite I, Vaitkus A (2019). Clinical and diagnostic features of patients with familial multiple sclerosis. Med Hypotheses.

[17] Koch M, Zhao Y, Yee I (2010). Disease onset in familial and sporadic primary progressive multiple sclerosis. Mult Scler.

[18] Romero-Pinel L, Martínez-Yélamos S, Gubieras L, Matas E, Bau L, Kremenchutzky M, Arbizu T (2010). Anticipation of age at onset in familial multiple sclerosis. Eur J Neurol.

[19] Tipirneni A, Weinstock-Guttman B, Ramanathan M (2013). MRI characteristics of familial and sporadic multiple sclerosis patients. Mult Scler.

[20] Siger-Zajdel M, Selmaj K (2001). Magnetisation transfer ratio analysis of normal appearing white matter in patients with familial and sporadic multiple sclerosis. J Neurol Neurosurg Psychiatry.

[21] Leary SM, Davie CA, Parker GJ (1999). 1H magnetic resonance spectroscopy of normal appearing white matter in primary progressive multiple sclerosis. J Neurol.

[22] Pytel V, Matías-Guiu JA, Torre-Fuentes L (2019). Exonic variants of genes related to the vitamin D signaling pathway in the families of familial multiple sclerosis using whole-exome next generation sequencing. Brain Behav.

[23] Tempest A, Veettil SK, Maharajan MK, Earl JC, Ngorsuraches S, Chaiyakunapruk N (2022). Genetic biomarkers in multiple sclerosis: an umbrella review of meta-analyses of observational studies. Mult Scler Relat Disord.

[24] Ebers GC, Koopman WJ, Hader W (2000). The natural history of multiple sclerosis: a geographically based study: 8: familial multiple sclerosis. Brain.

[25] Taul-Madsen L, Riemenschneider M, Jørgensen MK, Dalgas U, Hvid LG (2022). Identification of disability status in persons with multiple sclerosis by lower limb neuromuscular function - emphasis on rate of force development. Mult Scler Relat Disord.

[26] Bussas M, El Husseini M, Harabacz L (2022). Multiple sclerosis lesions and atrophy in the spinal cord: distribution across vertebral levels and correlation with disability. Neuroimage Clin.

[27] Nakamura Y, Liu Z, Fukumoto S (2020). Spinal cord involvement by atrophy and associations with disability are different between multiple sclerosis and neuromyelitis optica spectrum disorder. Eur J Neurol.

[28] Andelova M, Uher T, Krasensky J (2019). Additive effect of spinal cord volume, diffuse and focal cord pathology on disability in multiple sclerosis. Front Neurol.

